# M. Delpech on the Nature and Symptoms of Varicella

**Published:** 1846-04

**Authors:** 


*M. Delpech on the Nature and Symptoms of Varicella.*—The *Journal de Medicine* for January and February contains an elaborate article by M. A. Delpech on an epidemic of Varicella, observed at the Hôpital Necker, to which he was then interne (house physician) in the year 1844. The epidemic lasted three or four months, and was confined to the patients of one ward, peopled by infants and very young children. The results at which M. Delpech has arrived, after a careful and minute analysis of the phenomena presented by the disease during this epidemic, are similar to those which have been generally adopted in this country of late years. His researches, however, are valuable as corroborative evidence. The following analysis of the more important parts of M. Delpech's essay will convey a correct idea of the Necker epidemic, as also of the pathological views which M. Delpech so ably advocates.

There were no prodromes, properly speaking; the appearance of the vesicular eruption being merely accompanied, or slightly preceded, by a little fever, heat of skin, nausea, or vomiting. During the course of the disease fresh eruptions would take place, each being accompanied by a renewal of the same general symptoms. The fluid of the vesicles first became lactescent, and then dried, passing through these periods in from three to seven days. The disease, itself, however, often lasted much longer, owing to successive eruptions taking place. On many of the patients, instead of vesicles, there were pemphigoid bullæ, or the latter appeared simultaneously with the former. Not unfrequently, when the vesicles had arrived at the period of desiccation, the crusts remained moist, and the skin continued to secrete pus. The pemphigoid varicellæ were those which most frequently gave rise to the unfavourable occurrence.

The presence of the bullæ seemed to indicate a deeper and more chronic inflammation of the skin ; the pus secreted was generally serous. The suppurative diathesis which seemed to exist in these cases can scarcely, however, be considered the legitimate sequela of varicella.

The prognosis of varicella may be always favourable, as it is always a very benign disease ; indeed, it has perhaps never been fatal of itself. It may, however, be associated with variolous eruptions, and then the state of the patient sometimes proves alarming. On the other hand, a child whilst labouring under varicella, may be attacked with other severe disease, and thus be placed in a critical position. Thus, M. Delpech mentions having seen a child subject to convulsions, die from an attack which occurred whilst it was affected with single varicella. In so mild a disease, there is, of course, but little treatment required. In the great majority of cases, hygienic means alone are necessary to treat it, or rather to prevent its progress from its being intempestively arrested. Nearly the only medicinal agent indicated is a mild purgative at the end of the treatment. It modifies favourably the purulent predisposition when the latter exists, by repulsive and secretive depuration. When the suppurative diathesis exists, in addition to the use of mild purgatives, topical remedies will be found of use, such as an ointment composed of calomel, one part; lard, ten parts ; or sulphur or corrosive sublimate baths.

M. Delpech appears to think that the Necker epidemic of varicella originated spontaneously at the hospital, but that it extended itself in the ward by contagion ; for other and adjoining wards were completely free. Nevertheless, he was not able to propagate it by inoculation. Two children, in a ward where there was no eruptive disease, were inoculated with varicella fluid taken from a patient out of the hospital, but without success. This result is confirmatory of various recent experiments, and seems to establish a marked and total difference between varicella on the one hand, and cowpox and variolous disease on the other. M. Delpech is the more inclined to attribute the propagation of the disease in the Necker ward to contagion, from the circumstance of the children being attacked in groups, as if they had been exposed to the cause of the disease at the same epoch, although the length of their residence in the hospital was varied. After a careful and elaborate comparative analysis of the epochs at which the attacks occurred, and of all the data of these attacks, M. Delpech thinks he is warranted in concluding that the disease is transmitted anteriorly to the fifth day of invasion, and probably between the third and fifth. He also thinks that the incubations of varicella may be fixed at about twelve days. M. Delpech's analysis of his case is so minute, that it sometimes requires a strong effort of attention to follow him. His deductions, however, appear legitimately drawn, and the process by which he arrives at them may be offered as a model for the followers of the numerical system.

M. Delpech concludes his essay by a parallel between varicella and variola. He shows that the origin of varicella is much more recent than that of variola, and reminds his readers that, as long as variola was observed in its primitive intensity, unmodified by inoculation or vaccination, no author thought of confounding the two diseases. Vidus Vidius, the first who gave a good account of varicella, expressly states that it is a different disease to variola or rubeola, as the following sentence testifies :—Quam obrem non videntur tanquam tertia species morbillis et variolis hæ pustulæ adjiciendæ, sed satis est si ad phlyctenas referantur. Varicella has been erroneously confounded with variola by some modern authors, owing principally to the circumstance of the two diseases being occasionally met with at the same time, on the same individual, the one not excluding the other. The separate individuality of varicella, however, is proved,—by its existing in an isolated state, free from all variolous complication, both sporadically and epidemically—by the age of those whom it attacks, nearly exclusively children—by the perfect indifference with which it attacks all individuals, whether vaccinated or not, whether they have had variola or not, even when labouring under varioloid disease, or when recently convalescent from it—the complete absence of all modification in the manifestations of the affection in those who are or have been under the influence of variolous affections—the absence of all immunity conferred by varicella from variola or cowpox—the possible combination, and simultaneous developement of varicella and variola—the impossibility of variola transmitting or giving rise to varicella, or of varicella transmitting or giving rise to variola—the doubtful possibility of transmitting varicella by inoculation, notwithstanding the experiments of Willan, which have been disproved by all subsequent experimentalists—and, lastly, the decided and constant difference in the form and symptoms of the two diseases.

This elaborate essay certainly does M. Delpech great credit. It has been rewarded by the Monthyon prize—a yearly prize, given by the Faculty of Medicine of Paris, for the best memoir on the diseases of the past year, as observed in the Paris hospitals.—*Ibid.*

